# Engineering of lipid membranes asymmetrically functionalized with chondroitin sulfate†

**DOI:** 10.1039/d4fd00195h

**Published:** 2025-05-09

**Authors:** Teresa Rodríguez-García, Loretta Akakpo, Sadie L. Nickles, Ryan J. Schuck, Daiane S. Alves, Katherine G. Schaefer, Frederick A. Heberle, Gavin M. King, Francisco N. Barrera

**Affiliations:** a Department of Biochemistry & Cellular and Molecular Biology, University of Tennessee Knoxville USA fbarrera@utk.edu; b Department of Chemistry, University of Tennessee Knoxville USA; c Department of Physics and Astronomy, University of Missouri Columbia USA; d Department of Biochemistry, University of Missouri Columbia USA

## Abstract

One of the defining properties of the eukaryotic plasma membrane is the glycocalyx, which is formed by glycosylated lipids and proteins. The glycocalyx is arranged asymmetrically, as it is exclusive to the extracellular side of the membrane. Membrane asymmetry therefore includes both lipid and carbohydrate asymmetry, whereby the latter has the most skewed trans-leaflet imbalance. The glycocalyx plays a structural role that protects cell integrity and it also participates in mechanosensing and other cellular processes. However, our understanding of glycocalyx function is hampered by the lack of suitable model systems to perform biophysical investigation. Here, we describe the engineering of lipid bilayers that are chemically conjugated at the outer surface with one of the most abundant glycocalyx components, chondroitin sulfate (CS). Membranes were doped with a reactive phospholipid, which allowed thiol–maleimide conjugation of thiol-modified CS at the lipid headgroup. Our data show that we achieved CS conjugation of large unilamellar vesicles, supported lipid bilayers, and giant unilamellar vesicles. CS conjugation of vesicles allowed electrostatic recruitment of poly-l-lysine, which could recruit other CS-coated vesicles or CS in solution. Overall, we describe a simple and robust method for polysaccharide functionalization of vesicles which can be applied to gain new mechanistic understanding of the pathophysiological role of the glycocalyx.

## Abstract 2

A model system that mimics the glycocalyx: a lipid bilayer coated with chondroitin sulfate.

## Graphical abstract

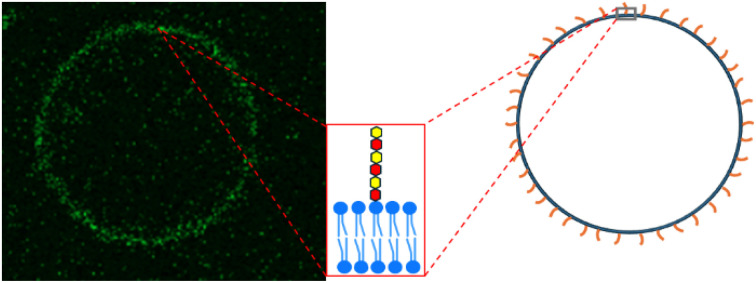

## Introduction

The plasma membrane of human cells is a highly complex and heterogeneous bilayer formed primarily by three families of molecules: lipids, proteins, and carbohydrates. The arrangement of these molecules in a specific pattern allows the membrane to be a multi-functional entity that is able to respond dynamically to changes in the cell microenvironment. A fundamental property of the plasma membrane is asymmetry, as the two lipid bilayer leaflets have drastically different molecular composition. Plasma membrane asymmetry primarily entails a strong gradient of two different molecule classes: (1) transversal lipid asymmetry refers to the large differences in lipid composition between the inner (cytoplasmic) and outer membrane leaflets,^1,2^ while (2) carbohydrate asymmetry occurs because glycosylated lipids and proteins are oriented in such a way that the sugar moieties face the cell exterior, constituting what is generally known as the glycocalyx.^3^ The glycocalyx forms a thick and dense matrix composed of a diverse array of glycosylated molecules: glycolipids, proteoglycans, mucins and glycoproteins.^4,5^ Far from merely working as a protective layer to the plasma membrane, the glycocalyx plays an active role that regulates a range of physiological and pathological processes.^4^ The cellular processes where the glycocalyx is involved include regulation of cell morphology,^6^ membrane protein diffusion,^7^ and the immune system.^8^ From a therapeutic standpoint, the glycocalyx also participates in target recognition by viruses,^9,10^ fungi^11^ and bacteria,^12,13^ and controls cancer cell development^14^ and can regulate cancer immunotherapy (checkpoint inhibition).^15^ Despite its importance, we have a limited understanding of glycocalyx functions, in part due to the lack of adequate model systems that allow quantitative investigation of the interplay between lipid and saccharide components.

Chondroitin sulfate (CS) belongs to the glycosaminoglycan family, and is one of the major components of the glycocalyx and the extracellular matrix. CS is a long (∼10–100 kDa) linear polysaccharide formed by repetition of a disaccharide unit:^4^ a d-glucuronic acid and a sulfated *N*-acetylgalactosamine.^16,17^ The different types of CS (A, C, D and E), vary on the position of the sulfation and epimerization.^17^ CS chains are linked to a core protein unit to form chondroitin sulfate proteoglycans (CSPG). CSPG are linked to the plasma membrane, as part of the glycocalyx, or linked at the extracellular matrix, where they participate in tissue formation. CSPG also regulate signaling by binding to growth factors, as they control their access to their target receptor.^18^

Two recent publications report on approaches to decorate vesicle with CS. Jahnke and co-workers chemically conjugated CS to cholesterol,^19^ and the soluble CS-cholesterol hybrid molecule is able to partition into the surface of vesicles. However, this strategy leads to cholesterol asymmetry in the plasma membrane, which is expected to strongly impact the physical properties of the membrane.^20,21^ Shioiri *et al.* used a synthetic approach to conjugate CS to the phospholipid phosphatidylethanolamine.^22^ While this is an elegant chemical approach, it requires precipitation and purification steps. An additional potential drawback of the two previous approaches, where the lipid-CS partitions into already formed vesicles, is the poor control over the final membrane density of the CS.

**Fig. 1:**
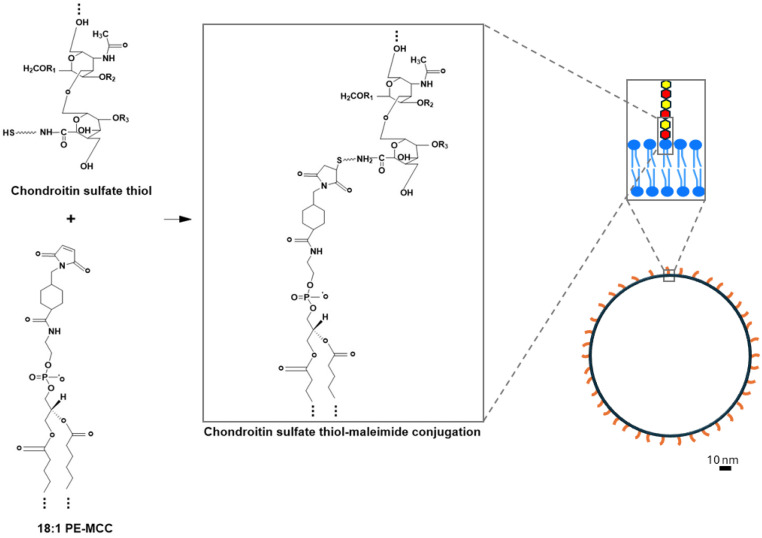
Conjugation of chondroitin sulfate thiol (CS) to a maleimide-modified lipid to form CS-functionalized large unilamellar vesicles. The chemical structure of CS and 18 : 1 PE-MCC is shown before and after thiol–maleimide bond formation. In the CS chemical structure, R1, R2, R3 = H or SO_3_H, depending on the type of CS subtype (A, C, D, or E) and the squiggly line indicates a spacer linker. Only part of the fatty acid chains and CS polymer are shown (marked with ellipsis). d-glucuronic acid and sulfated *N*-acetylgalactosamine are shown as red and yellow hexagons. The figure is approximately to scale.

Here, we report a facile method to form lipid bilayers that are coated with CS (Fig. 1). Our data show that this approach generates CS-functionalized bilayers for the three most common types of model membranes: large unilamellar vesicles (LUVs), supported membranes, and giant unilamellar vesicles (GUVs).

## Methods

### Reagents

The lipids POPC (1-palmitoyl-2-oleoyl-*glycero*-3-phosphocholine) (Catalog No. 850457), 18 : 1 PE-MCC (1,2-dioleoyl-*sn*-glycero-3-phosphoethanolamine-*N*-[4-(*p*-maleimidomethyl) cyclohexane-carboxamide] (sodium salt)) (Catalog No. 780201), DOPC (1,2-dioleoyl-*sn*-glycero-3-phosphocholine) (Catalog No. 850375), 18 : 1 PE-TopFluorAF594 (1,2-dioleoyl-*sn*-glycero-3-phosphoethanolamine-*N*-(TopFluorAF594) (ammonium salt)) (Catalog No. 810387), and 18 : 1 NBD PE (1,2-dioleoyl-*sn*-glycero-3-phosphoethanolamine-*N*-(7-nitro-2-1,3-benzoxadiazol-4-yl)) (ammonium salt) (Catalog No. 810145) were purchased from Avanti Polar Lipids, Alabaster, AL. Tris base (2-amino-2-(hydroxymethyl)-1,3-propanediol) (Catalog No. BP152-500) and Wisteria Floribunda Lectin-FITC Labeled (WFA) (Catalog No. L32481) were purchased from Fisher Scientific. We used a mixture of chondroitin sulfate A, C, D and E that was functionalized with a thiol group from Haworks LLC, Bedminster, NJ (CS-Thiol-25k), which is abbreviated simply as CS. Poly-l-lysine (PLL) labeled with the fluorophore FITC (Catalog No. P3543-10MG) was purchased from Millipore Sigma, Burlington, MA.

### CS conjugation to large unilamellar vesicles

POPC and 18 : 1 PE-MCC stocks were prepared in chloroform. Aliquots of lipids with 99.5 mol% POPC and 0.5% 18 : 1 PE-MCC were dried under argon gas and then placed in a vacuum overnight. Lipid films were resuspended in 10 mM Tris, 350 mM NaCl buffer, pH 7.5. Large unilamellar vesicles (LUVs) were formed by extrusion with a Mini-Extruder (Avanti Polar Lipids, Alabaster, AL) through a 100 nm pore size membrane (Whatman, United Kingdom) at room temperature. Functionalization of the LUVs was performed with CS prepared in the same buffer at a 2 : 1 CS : lipid molar ratio. The conjugation reaction with CS was incubated for 2 hours with constant shaking at room temperature.

### NBD head group environment fluorescence assay

LUVs conjugated with chondroitin sulfate were prepared as described above, but in this assay the lipid composition of the LUVs was 98.5 mol% POPC, 1% 18 : 1 NBD PE, and 0.5% 18 : 1 PE-MCC. Samples were loaded into a black 96-well plate (Corning, Kennebunk, ME) to measure the fluorescence spectra on a Cytation 5 plate reader (BioTek, Winooski, VT), using an excitation wavelength of 460 (±10) nm and an emission wavelength of 535 (±10) nm.

### Dynamic light scattering (DLS)

We used a Dynapro Nanostar I DLS instrument (WYATT Technology, Santa Barbara, CA), which uses a 658 nm laser at a controlled temperature of 25 °C. The scattered light was measured at an angle of 90°. For the analysis, the LUVs were prepared as described in the previous paragraph. A titration with FITC-conjugated poly-l-lysine (PLL-FITC) was performed after a 30 minutes incubation. CS and PLL controls were also prepared. All samples were diluted with 10 mM Tris, 350 mM NaCl buffer, pH 7.5 for the corresponding final concentrations, and to a final volume of 100 μL. The samples were measured into a cyclic olefin copolymers cuvette (WYATT Technology, NC0616299). All DLS data were processed using the DYNAMICS software (WYATT Technology, Santa Barbara, CA), and the size distribution is shown considering the mass particle size distribution.

### Removal of unconjugated chondroitin sulfate

To remove the unconjugated chondroitin sulfate of the glycoliposome samples, five washings of the PCMal-CS *via* centricon (Vivaspin 6, 100 kDa; Millipore Sigma, Catalog No. GE28-9323-19) were performed. Glycoliposomes were prepared as stated in the “CS conjugation to large unilamellar vesicles” section. After the conjugation with CS, five centrifugation steps (3260×*g*, 10 minutes, 25 °C) were done. Flow through samples of every washing were collected in order to measure the concentration of CS after each one. To perform this measurement, we used the lectin WFA labeled with FITC. WFA binding to CS causes a fluorescence increase, allowing to create a calibration curve where the fluorescence intensity of the FITC labeled in WFA increases as the [CS] increases. Samples were loaded into a black 96-well plate (Corning, Kennebunk, ME) to measure the fluorescence spectra on a Cytation 5 plate reader (BioTek, Winooski, VT), using an excitation wavelength of 495 (±9) nm and an emission wavelength of 515 (±9) nm. In each replicate, a linear calibration curve was created at the same time as the flow through samples were measured. After the centrifugation, the PCMal-CS sample was measured using DLS.

### Lipid preparation for AFM

DOPC and 18 : 1 PE-MCC at a 99.5 : 0.5 molar ratio, were dried in glass culture tubes, back-filled with argon, sealed, and stored at −20 °C. At the time of extrusion, room-temperature phosphate-buffered saline (PBS) was added to swell the lipids. The lipid solution was extruded (Liposofast, Avestin) through 100 nm membranes 51 times, to form unilamellar vesicles. The solution was aliquoted and stored at −80 °C until the time of the AFM experiments. On the day of experiments, CS was incubated with the DOPC PE-MCC lipid in a 2 : 1 CS : lipid molar ratio for 1 hour with constant shaking.

### Supported lipid bilayer formation and AFM imaging

80 μL of the sample solution was deposited on a freshly cleaved mica disc and incubated for 10 min at room temperature to allow for adhesion to the surface. Loosely bound material was washed away *via* buffer exchange (80 μL of PBS, 5–6 times). All images were acquired in PBS using biolever mini tips (Bruker, *k* ∼ 0.1 N m^−1^, *F*_0_ ∼ 25 kHz in fluid) on a Cypher, Asylum Research AFM in tapping mode. Care was taken to keep the tip-sample force <100 pN. Analysis was performed using commercial software (Asylum Research, Inc).

### GUVs formation and functionalization

GUVs were electroformed in the presence of 100 mM sucrose using an analog heat block connected to a function generator. 250 nanomoles of lipid (97 mol% POPC, 2% 18 : 1 PE-MCC and 1% PE-TopFluorAF594) dissolved in 200 μL of chloroform, were spread homogeneously on the conductive site of indium-tin-oxide (ITO) coated glass slides (Delta Technologies, Loveland, CO). Subsequently, the lipid-film-coated ITO slides were desiccated for 2 hours by placing the slides in a heated chamber (55 °C) attached to a vacuum pump, to remove the remaining chloroform. An O-ring spacer was placed on top of the lipid-coated ITO slide, which was then filled up with 600 μL of 100 mM sucrose, 10 mM Tris, pH 7.5 solution. A sealed chamber was created by placing a second ITO slide on top. Electrodes were connected with the conductive sides of the ITO slides and the preinstalled standard program was run, generating an AC field of 2 V and 10 Hz for 2 h at 30 °C.

After electroformation, GUVs were functionalized with CS. For the conjugation, 70 μL of GUVs were incubated for 1 hour with 70 μL of CS 25 μM, in 10 mM Tris, 100 mM sucrose, pH 7.5 buffer. GUVs were then incubated with 0.5 μM PLL-FITC for 30 minutes. GUVs conjugated with chondroitin in the presence of PLL-FITC were harvested by sedimentation after 20 minutes in 1 mL of 100 mM glucose, 10 mM Tris, pH 7.5 solution. Subsequently, they were placed on a slide with a spacer between slide and coverslip for confocal imaging.

### Confocal microscopy imaging of GUVs

Confocal microscopy was performed on an inverted Zeiss LSM 900/Airyscan laser scanning confocal microscope (Carl Zeiss AG). The pinhole was set to 1 airy unit, and all experiments were conducted at room temperature. For image acquisition, a 63X oil immersion objective (HCPL APO 63X/1,40 OIL CS2) was used. The rAF594 channel used an excitation wavelength of 561 nm and the FITC channel of 488 nm. Images were analyzed and adjusted in ZEISS ZEN 3.11 software.

## Results

We sought to develop a straightforward approach for covalent conjugation of CS to lipid membranes. We employed the thiol–maleimide reaction, commonly used for protein labeling, and applied it in this case to create a hybrid lipid molecule with a long and negatively charged CS chain at the headgroup (Fig. 1, left). To this end, we employed two commercially available reagents: a 25 kDa chain of CS that was modified with a thiol moiety, and the lipid 18 : 1 PE-MCC, which we will refer to as PCMal, where a maleimide group is incorporated at the phosphatidylethanolamine headgroup. Incubation at room temperature and neutral pH leads to formation of a thiol–maleimide covalent bond.

**Fig. 2:**
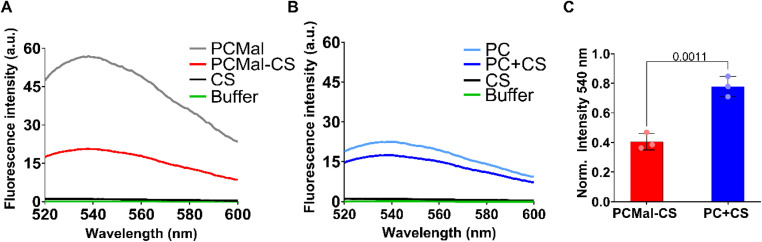
NBD headgroup environmental assay. Representative fluorescence spectra for CS incubation with PCMal (A) or non-reactive PC (B) LUVs. Control fluorescence for buffer and CS in buffer are shown. (C) Quantification of NBD fluorescence intensity at 540 nm are shown for PCMal-CS and for PC + CS, normalized to conditions in the absence of CS. The error bars are the S.D., and the *p* value was obtained from a one-tailed Student’s *t*-test (*n* = 3).

We expected that the presence of the negatively charged CS chain would increase the polarity of the lipid headgroup layer. We devised an assay to test the success of the conjugation, by incorporating an environmentally-sensitive reporter at the lipid headgroup. We chose an NBD fluorophore incorporated into a phospholipid headgroup, as the fluorescence of NBD decreases in a more polar medium.^23–27^ To perform this assay, we formed POPC large unilamellar vesicles (LUVs) that contained 0.5 mol% of PCMal and 1 mol% of an NBD-labeled phospholipid. We allowed these vesicles to react with thiol-modified CS (for simplicity we will refer to this reagent simply as CS hereafter). We observed a large change in the NBD fluorescence spectra (Fig. 2A), characterized by an important decrease in fluorescence intensity (Fig. 2C). To ensure that this fluorescence change resulted from CS conjugation to the LUVs, we performed control experiments where PE-MCC was not present in the vesicles. In this case we observed only a small change in fluorescence (Fig. 2B and C), which might result from non-covalent interaction of CS with the bilayer. The larger fluorescence intensity change observed with PCMal suggests that there is covalent attachment of CS to PCMal LUVs.

**Fig. 3:**
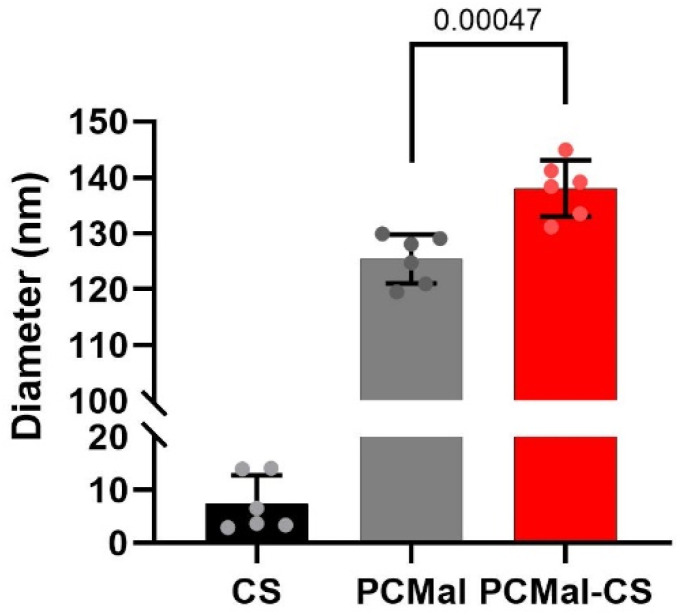
DLS study of LUV size. DLS data are shown for thiol-modified chondroitin sulfate (CS) in solution, LUVs containing maleimide-reactive PE-MCC lipid (PCMal), and for PCMal LUVs after conjugation with CS (PCMal-CS). Error bars are the S.D., and the *p* value was obtained from a one-tailed Student’s *t*-test (*n* = 6).

The 25 kDa CS chains that we employed contain more than 50 disaccharide units. Therefore, after they are conjugated to the LUVs, they are expected to cause a detectable increase in vesicle hydrodynamic radius (Fig. 1, right). We employed dynamic light scattering (DLS) to investigate vesicle size. Control DLS experiments with free CS showed that the polysaccharide particle has an effective diameter of 7.5 ± 5.3 nm (mean ± S.D.) (Fig. 3 and ESI Fig. 1†), suggesting that the CS chains are partially elongated in solution. We performed DLS before and after conjugation, and observed an increase in vesicle size of ∼15 nm (Fig. 3), compatible with CS-coated LUVs (Fig. 1). This result supports that the conjugation was successful.

Since we performed the maleimide-thiol conjugation with a molar excess of CS, we expect free CS to be found outside the vesicles. We performed serial centrifugal concentration steps using a Centricon with a 100 kDa bilayer, which is expected to allow free CS to go into the flow-through (FT), while CS-coated LUVs remain inside the centrifugal device. After each step, fresh buffer was added and the process was repeated a total of five times, where we expect a serial dilution to occur in each FT. We determined the CS levels in the FTs by quantification with a lectin that binds CS, conjugated to a fluorophore. Specifically, we used the *Wisteria floribunda* (WFA) lectin labelled with fluorescein. As expected, we observed a sequential decrease is free CS in each centrifugal step, and that five steps were enough to remove most CS (ESI Fig. 2†). The vesicle size did not change significantly after CS removal. However, we observed the appearance of an unexplained particle population of ∼25 nm. Therefore, we performed the rest of the experiments without the centrifugal step. Next, we studied the colloidal stability of the PCMal-CS vesicles. After the conjugation we stored the samples at 4 °C, and performed DLS experiments over two weeks. The data showed no major change in vesicle diameter over time (ESI Fig. 3†), suggesting that the CS-conjugated LUVs are stable long-term under refrigerated storage. Finally, we tested the effect of a −80 °C freeze/thaw cycle. DLS was performed after thawing, and we observed no diameter change (ESI Fig. 3†). These DLS results suggest that our protocol for coating LUVs with CS yields robust vesicles that can be stored at 4 and −80 °C. These results underscore the ease of experimentation with these vesicles, which do not require daily preparation and can be reused after −80 °C storage.

**Fig. 4:**
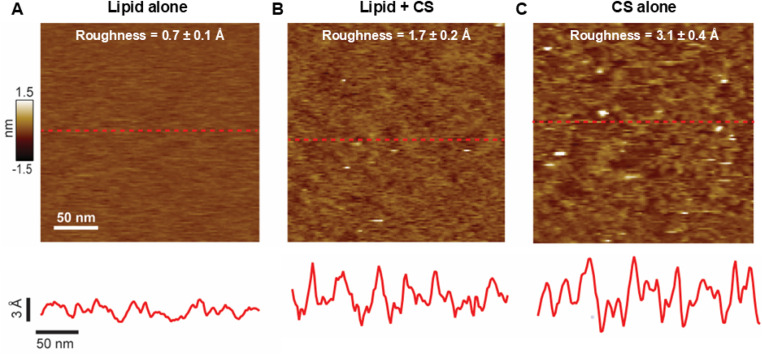
Chondroitin sulfate conjugation increases the roughness of the lipid bilayer surface. (A) AFM image of DOPC in the absence of CS. The root mean square roughness (mean ± standard deviation) is displayed on top. The dashed red line identifies a line scan that is displayed below the image. (B) Data for DOPC PCMal lipid conjugated with CS. A greater than 2-fold increase in bilayer surface roughness is observed over bare DOPC. (C) Chondroitin sulfate deposited directly on mica (in the absence of lipid). All data was acquired in aqueous buffer solution at room temperature.

The use of bilayers deposited on solid supports, forming supported lipid bilayers (SLB), is a popular method that allows the use of different types of microscopy to study lipid membranes. We applied our protocol for CS functionalization to SLB, which were imaged using atomic force microscopy (AFM). We employed membranes of DOPC, the lipid of choice for AFM due to the ease of spreading onto the underlying mica surface,^28,29^ doped with PCMal. Fig. 4 displays AFM images showing that conjugation with CS causes changes in membrane morphology. As expected, pristine bilayers were largely homogeneous and flat (Fig. 4A), while bilayers that were subjected to CS conjugation followed by extensive rinsing, showed heterogeneities on top of the bilayer plane (Fig. 4B and ESI Fig. 4†).

We estimated surface homogeneity by quantification of the root mean square roughness in 4 non-overlapping 100 nm × 100 nm regions. The lipid-only bilayer surface was smooth, with a roughness of less than 1 Å. However, after conjugation with CS, the roughness approximately doubled (Fig. 4 and ESI Fig. 4†). Line analysis illustrates local increases in height of 5–10 Å that are compatible with conjugation of CS molecules that lay mostly flat on the lipid membrane. Under these conditions, we expect the CS chains not to be perpendicular to the SLB surface, as they are highly flexible and could be deflected downward by the AFM tip, unlike the DLS experiments, where they are expected to be more extended on the surface of the LUVs (Fig. 1, right), hence the significantly larger dimensions observed by DLS (compare Fig. 3 and 4B). Finally, we performed a control experiment where CS was deposited directly on the mica, in the absence of lipid. As expected, these data show roughness (Fig. 4C) due to the polysaccharide. This control experiment supports the notion that membrane roughness originates from CS functionalization. Taken all together, the AFM results indicate that conjugation of CS to the membrane results in a molecularly rough surface. This result is expected for the low (0.5 mol%) maleimide lipid density that we employed. In these conditions the anchor points of CS to the membrane, if one considers that they are distributed randomly in the membrane, would be separated by ∼10 nm (Fig. 1, right).

**Fig. 5:**
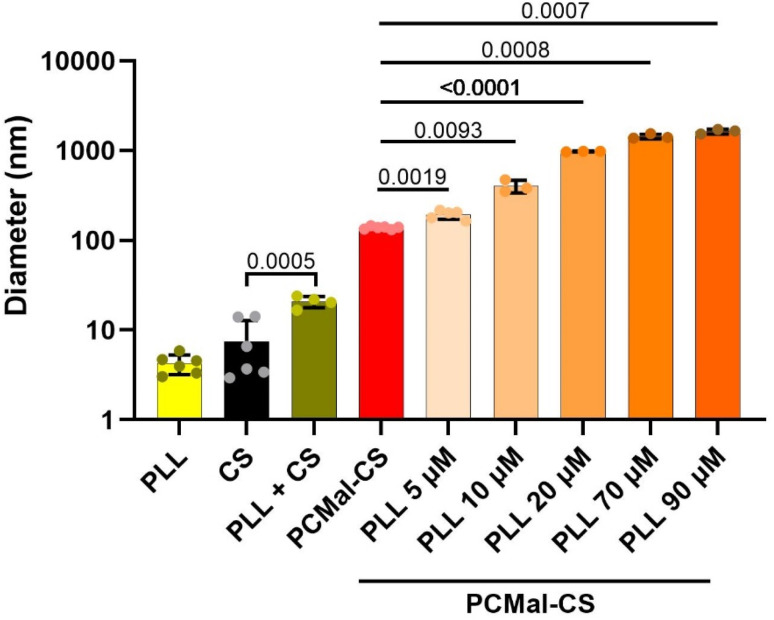
Poly-l-lysine crosslinks PCMal-CS LUVs. Control DLS data are shown for Poly-l-lysine-FITC (PLL), CS, and CS incubated with PLL. PCMal-CS LUVs were incubated with increasing concentrations of PLL and DLS measurements performed. The error bars are S.D., and *p* values were obtained from a one-tailed Student’s *t*-test (*n* = 3–6).

CS coating of the lipid is expected to change the physical properties of the vesicles, and allow for the introduction of new functionalities. As a simple test, we studied the interaction with poly-l-lysine (PLL). PLL is a synthetic polypeptide that is often used in cell culture experiments to provide a favorable substrate for human cells to grow on a plastic surface. PLL favors cell spreading and attachment as it establishes favorable electrostatic interactions with negative charges in the glycocalyx.^16,30^ We chose a FITC-labeled and long PLL chain (24 kDa), reasoning that after binding to the surface of a CS-coated LUV, the PLL polymer might protrude and interact with a second LUV, in practice non-covalently crosslinking different vesicles. We tested this hypothesis incubating PCMal LUVs with PLL. Control DLS experiments showed that PLL interacted with free CS (Fig. 5), forming a complex that was larger (20.7 ± 3.0 nm; mean ± S.D.) than the addition of the sizes of the PLL and CS particles. This result indicates that PLL interacts electrostatically with sulfate groups on CS. When we added PLL to the PCMal-CS LUVs, we observed a magnified effect. Fig. 5 shows DLS data for CS vesicles incubated with increasing concentrations of PLL (ESI Fig. 5†). The diameter of the particles increased several fold, up to a size of ∼3 μm (note that the vertical axis uses a logarithmic scale). These results suggest agglomeration of vesicles, showing that CS is able to endow vesicles with new functionality.

**Fig. 6:**
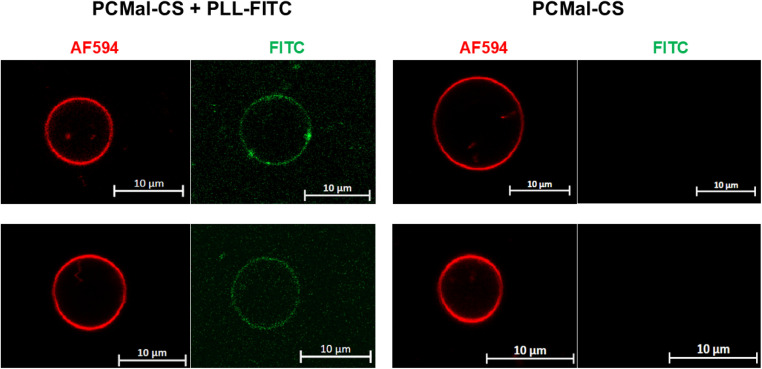
Giant unilamellar vesicles functionalization with CS. Left panel, PCMal-CS GUVs were incubated with PLL-FITC. The red channel shows the AF594 lipid, and the green channel PLL-FITC. Right panel, control experiment in the absence of PLL-FITC. Two GUVs are shown for each experimental condition. The equatorial variation in fluorescence intensity of AF594 is likely due to a polarization effect. Scale bars are 10 μm.

Giant unilamellar vesicles (GUVs) are cell-sized liposomes that have become the standard membrane model system for confocal microscopy investigation of lipid membranes.^31^ We tested if our thiol–maleimide conjugation scheme was able to achieve CS conjugation of GUVs. We used a standard electro-swelling protocol to form GUVs, which was followed by incubation with CS. GUV visualization was achieved by vesicle doping with the fluorescent lipid TopFluor-A594. Since the DLS data indicated binding of PLL-FITC to CS-conjugated vesicles, we used this reagent for fluorescence detection of CS on the GUVs. However, since GUVs have low mechanical resistance, we used different experimental conditions that were geared towards avoiding potential GUV disruption due to vesicle agglomeration. Therefore, we used a low GUV density, where the probability of two vesicles interacting with the same PLL-FITC molecule are expected to be low. Fig. 6 shows data collected after CS conjugation in the presence (left) or absence (right) of PLL-FITC. As expected, we observed GUVs larger than 5 μm in diameter, with a range of sizes. We observed that PCMal-CS GUVs were covered with CS, as reported by green labeling due to PLL-FITC recruitment. We observed that GUVs were frequently decorated with bright green spots. Since we use a molar excess of CS, we expect CS to be found in solution. Our images contained green particles on the vesicles (Fig. 6), which we attribute to free CS that is aggregated by PLL-FITC. We propose that the green spots observed on the vesicle surface result from PLL recruitment of these particles into the GUVs, analogous of how CS crosslinked LUVs (Fig. 5).

## Conclusions

This work describes a facile new method to functionalize model membranes with long CS polymers. Our approach uses the 18 : 1 PE-MCC (PCMal) reactive lipid, which contains a maleimide group in the headgroup that is able to form a covalent bond with thiol-modified CS. Both reagents are commercially available, as well as for alternate lipid and glycosaminoglycan options. Such conjugation created a synthetic glycolipid that remained anchored to the membrane due to the hydrophobicity of the acyl chains. We demonstrate the success of the approach in the three most common types of reconstituted membranes: LUVs, GUVs and SLBs.

We performed the covalent linkage after membrane formation to minimize impact of the CS on the different membrane model systems that we employed. This approach is expected to yield strongly asymmetric membranes with a skewed CS distribution. The SLBs we formed will have no lipid acyl chain asymmetry, since the acyl chains of the DOPC and PCMal lipids are identical. However, the LUVs were formed with POPC, and therefore we expect some asymmetry in the distribution of the palmitoyl acyl chain between the inner and outer membrane leaflet. We expect that the rate of trans-bilayer flip-flop of asymmetric glycolipid to be extremely slow,^32^ due to the large size (25 kDa) and multiple negative charges of the CS chain. Therefore, we expect CS asymmetry to be maintained for long periods of time.

The strategy that we present for functionalization of lipid membranes with a polysaccharide is straightforward and can be easily carried out in most biochemistry laboratories. Unlike previous methods for lipid modification with polysaccharides,^14,19,22,33^ our approach does not require complex chemical synthesis or purification of modified lipid species. We observed that our CS-liposomes were stable at least over a period of two weeks, and were not significantly disrupted by a freeze–thaw cycle, underscoring their ease of use and robustness as a new model system.

Functionalization of lipid vesicles with glycosaminoglycans like CS allows building membrane complexity in a systematic manner and provides a pathway towards creating reconstituted glycocalyx model systems with near-native characteristics. The glycocalyx is a dense medium where glycoproteins and glycopolymers are heavily entangled. As a first step towards reproducing this scenario, we used PLL for non-covalent crosslinking of CS. Our data show that our method allows association between vesicles (Fig. 5) and non-covalent recruitment of CS chains into the membrane (Fig. 6). Potential future applications for this technology include the development of membrane-based biosensors, biophysical investigations of the glycocalyx, stealth drug delivery, and the creation of more chemically complex artificial cells.

## Conflicts of interest

There are no conflicts to declare.

## Supplementary Material

FD-259-D4FD00195H-s001

FD-259-D4FD00195H-s002

FD-259-D4FD00195H-s003

FD-259-D4FD00195H-s004

FD-259-D4FD00195H-s005

FD-259-D4FD00195H-s006

## Data Availability

Data are available upon request from the authors.
