# Supplementary figures and images for: Engineering of lipid membranes asymmetrically functionalized with chondroitin sulfate
Source: Faraday Discuss. 2025 May 9;259:168–81. doi: 10.1039/d4fd00195h (PMC12062790; doi:10.1039/d4fd00195h)

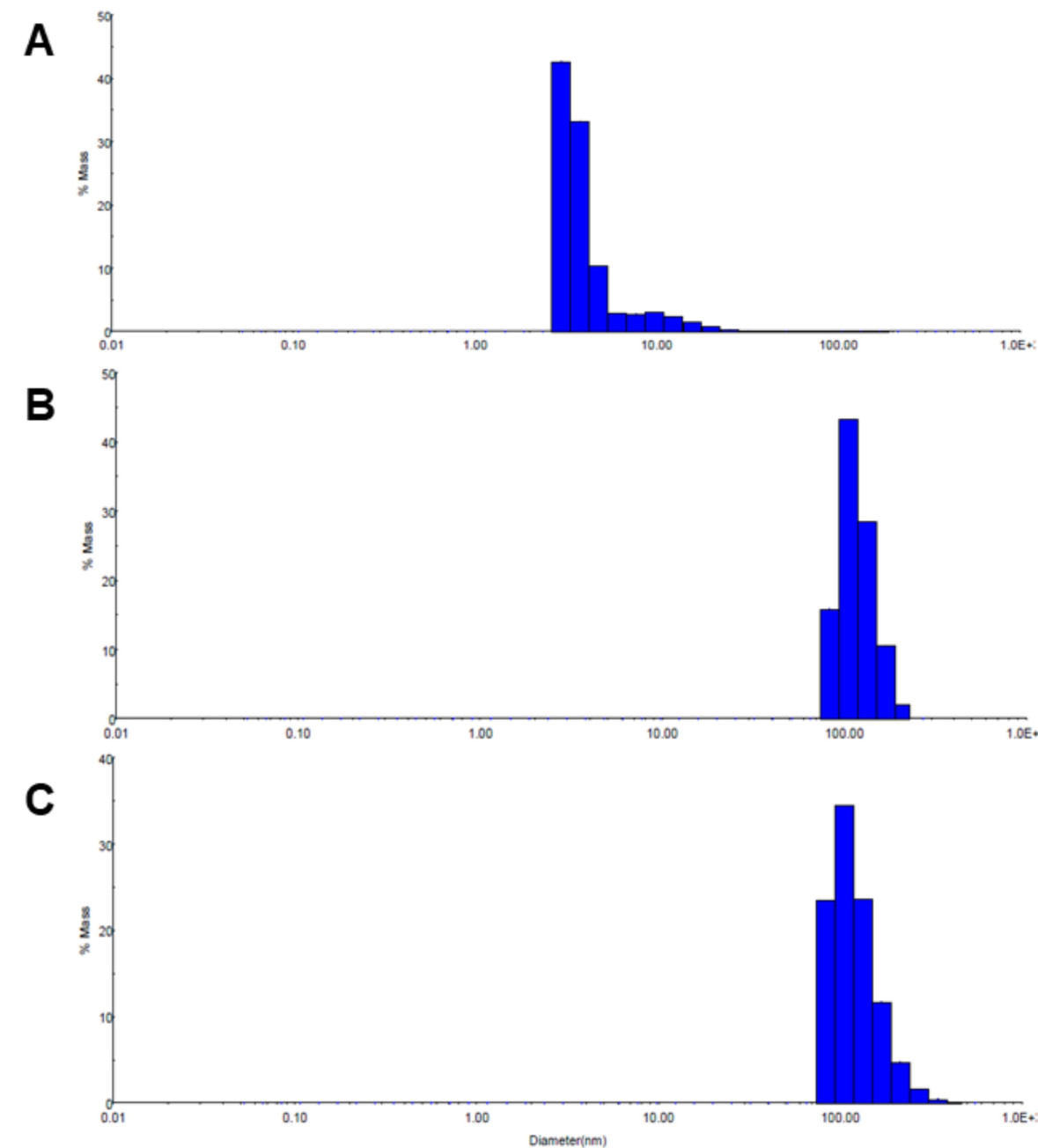

Supplement: FD-259-D4FD00195H-s001 [file FD-259-D4FD00195H-s001.pdf]

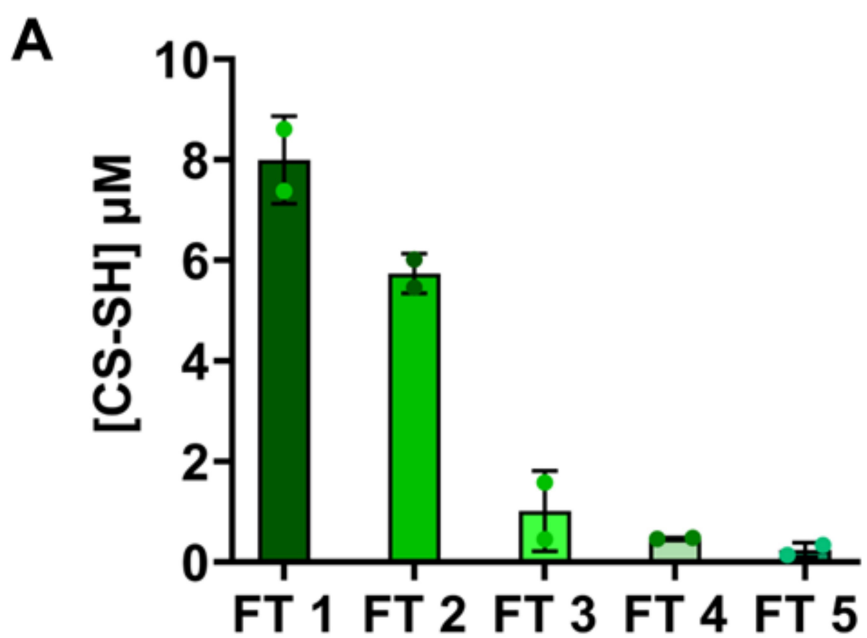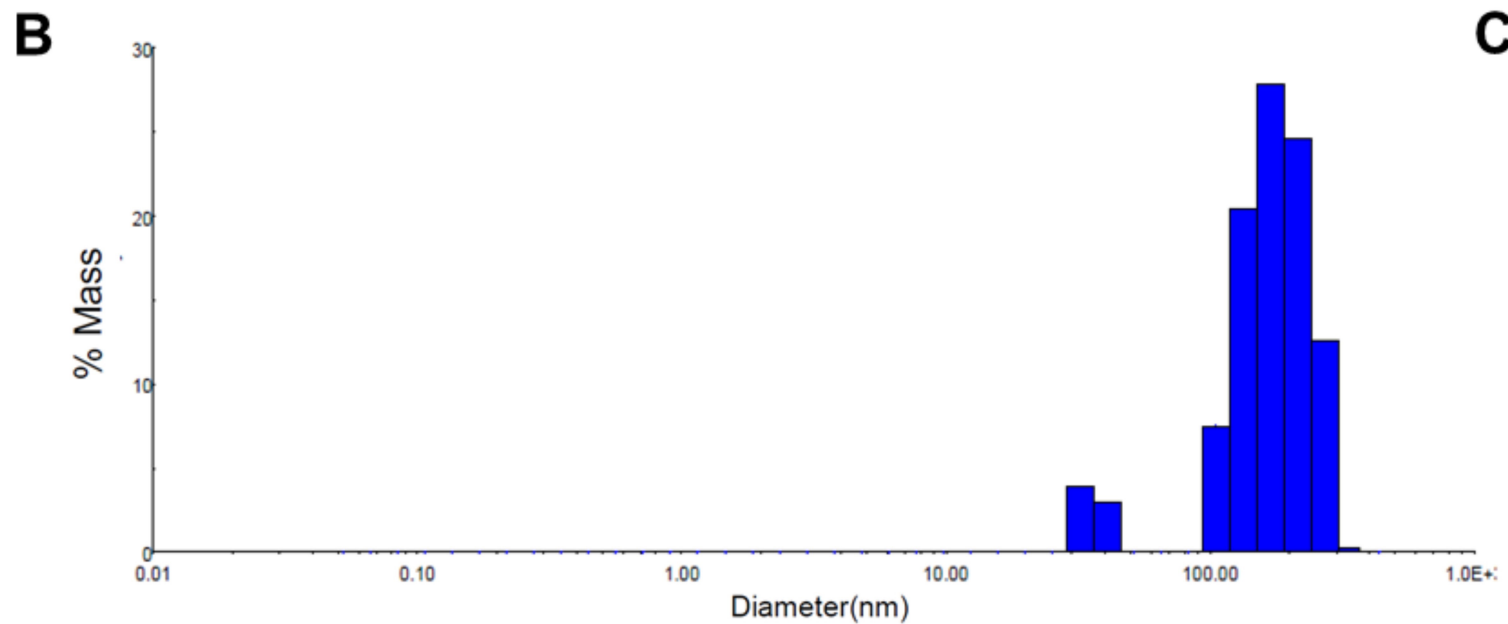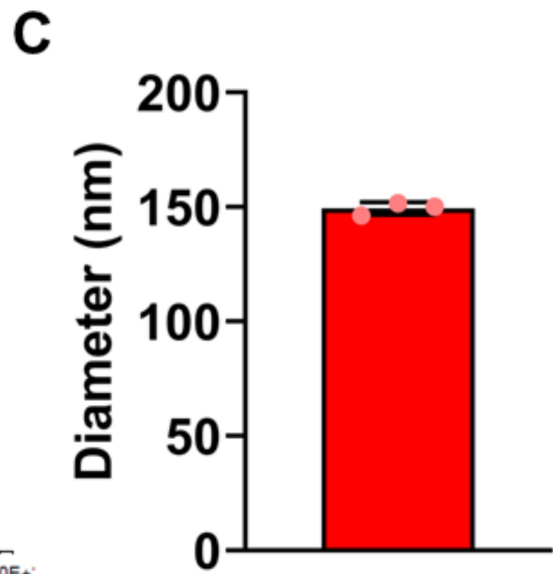

Supplement: FD-259-D4FD00195H-s002 [file FD-259-D4FD00195H-s002.pdf]

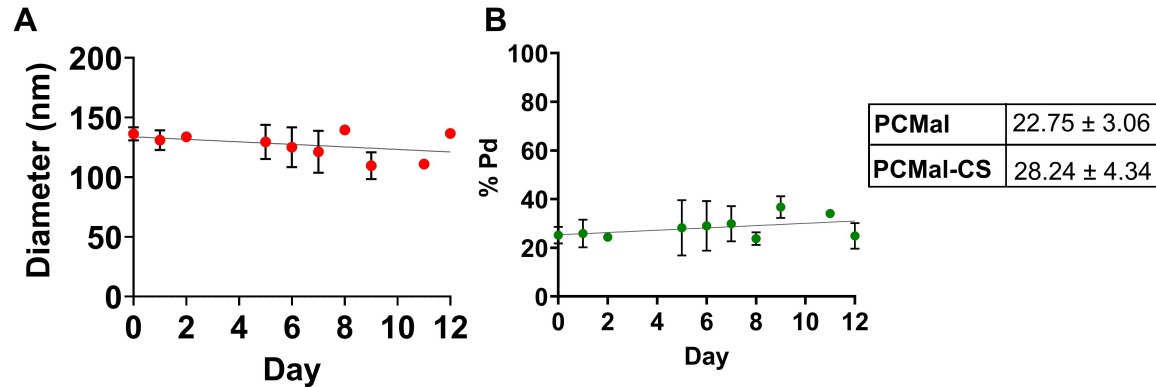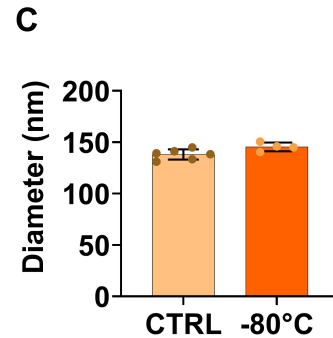

Supplement: FD-259-D4FD00195H-s003 [file FD-259-D4FD00195H-s003.pdf]

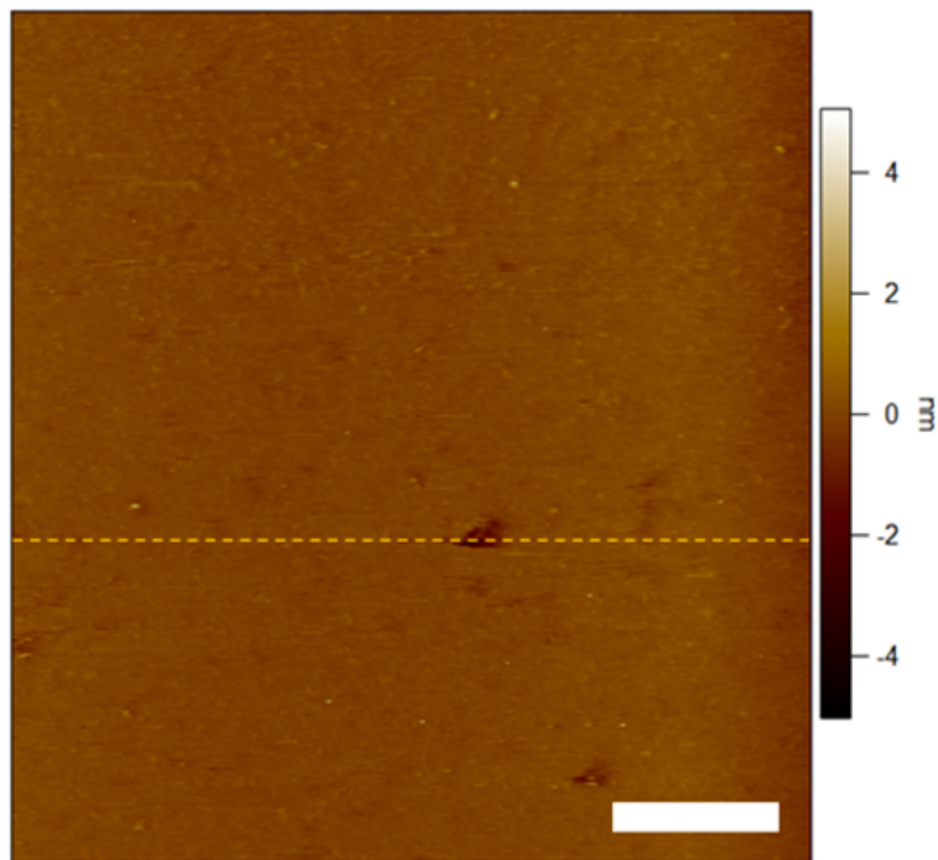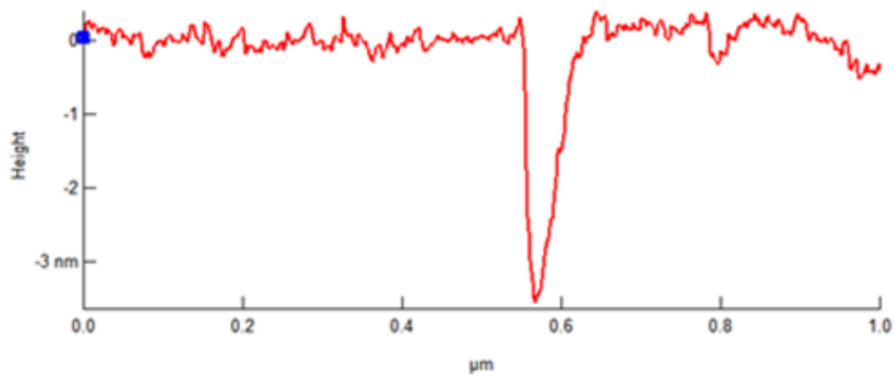

Supplement: FD-259-D4FD00195H-s004 [file FD-259-D4FD00195H-s004.pdf]

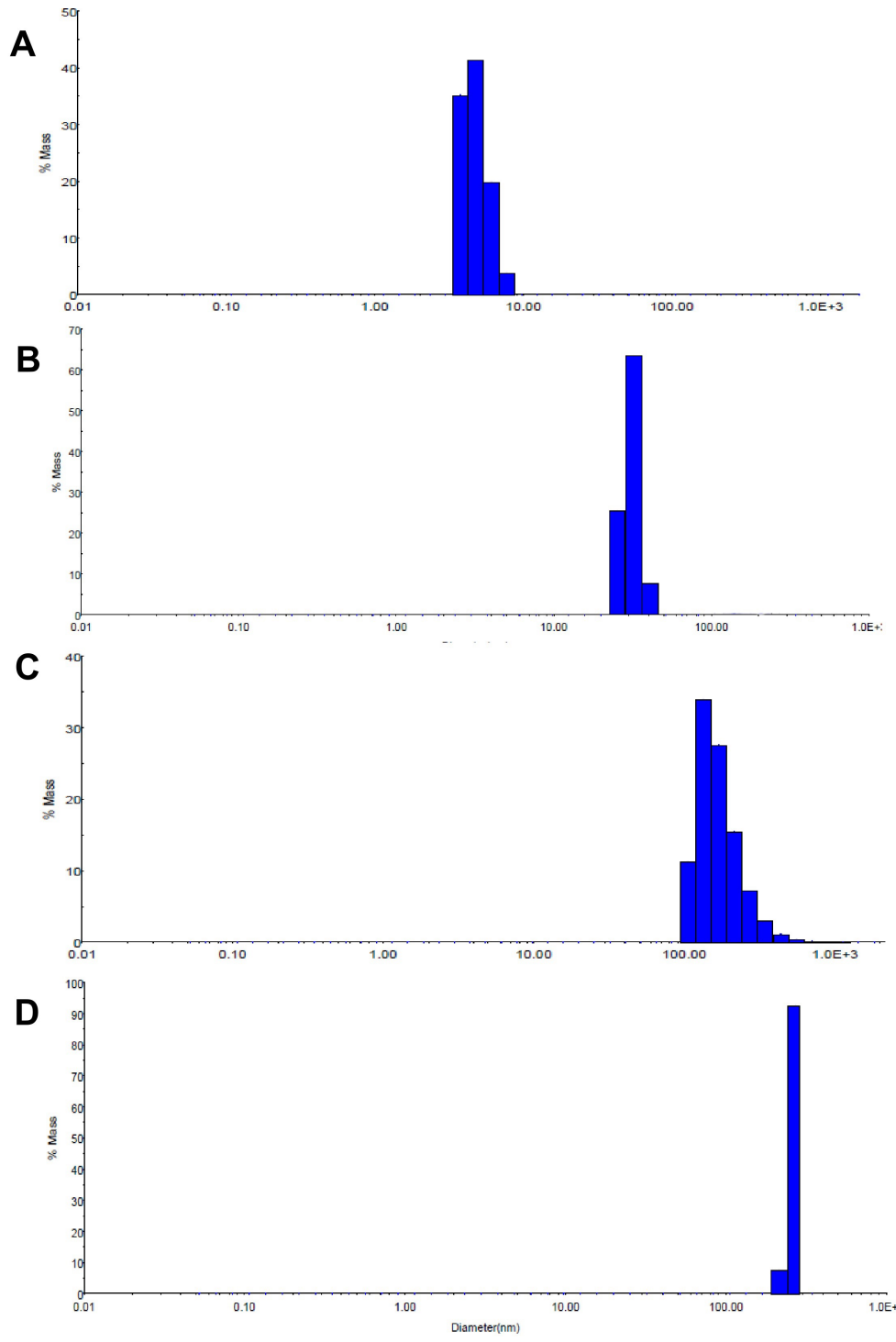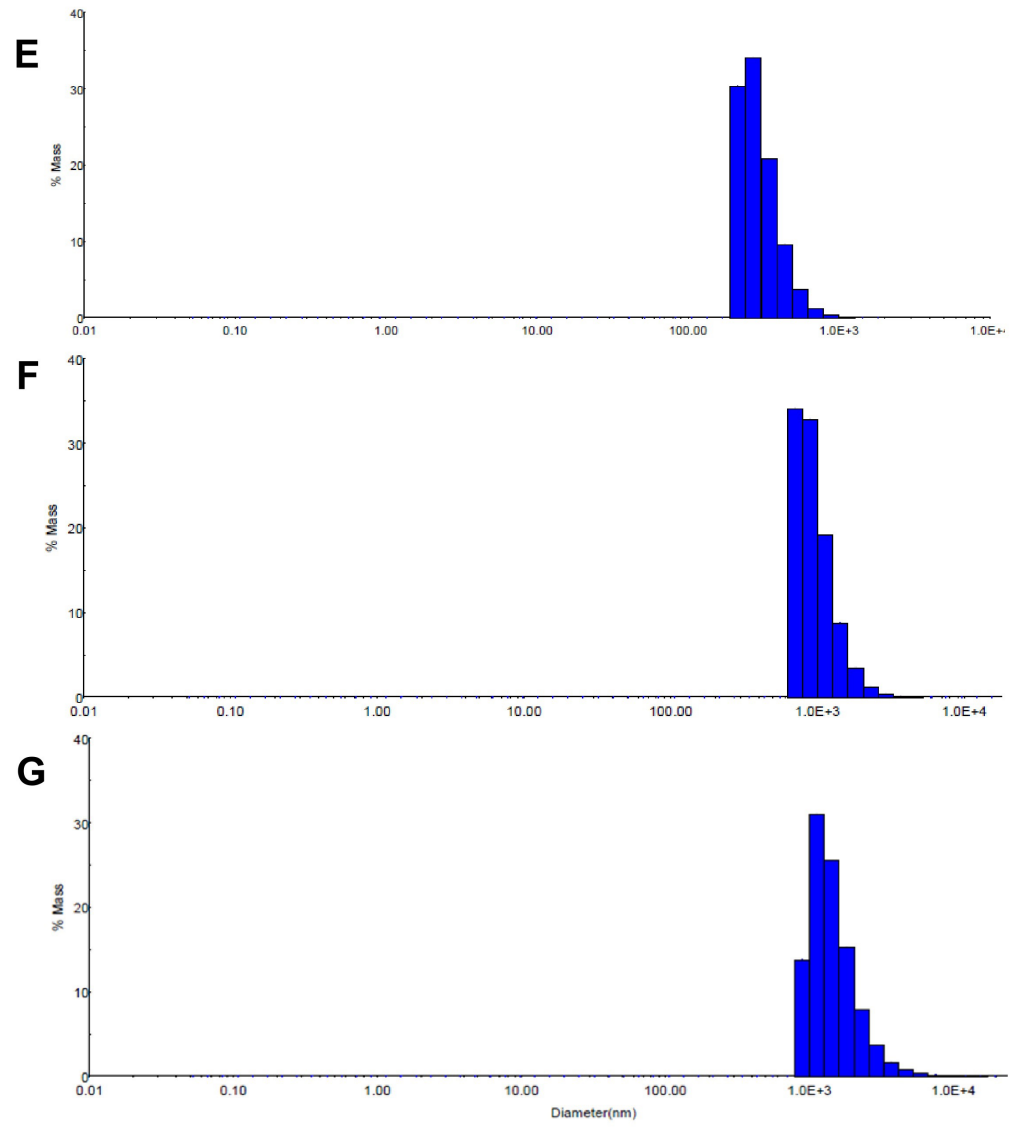

Supplement: FD-259-D4FD00195H-s005 [file FD-259-D4FD00195H-s005.pdf]
