# Supplementary material for: Engineering of lipid membranes asymmetrically functionalized with chondroitin sulfate
Source: Faraday Discuss. 2025 May 9;259:168–81. doi: 10.1039/d4fd00195h (PMC12062790; doi:10.1039/d4fd00195h)
Supplement: FD-259-D4FD00195H-s006 [file FD-259-D4FD00195H-s006.pdf]

Supplementary Information (SI) for Faraday Discussions.
This journal is © The Royal Society of Chemistry 2025

Supplementary Materials

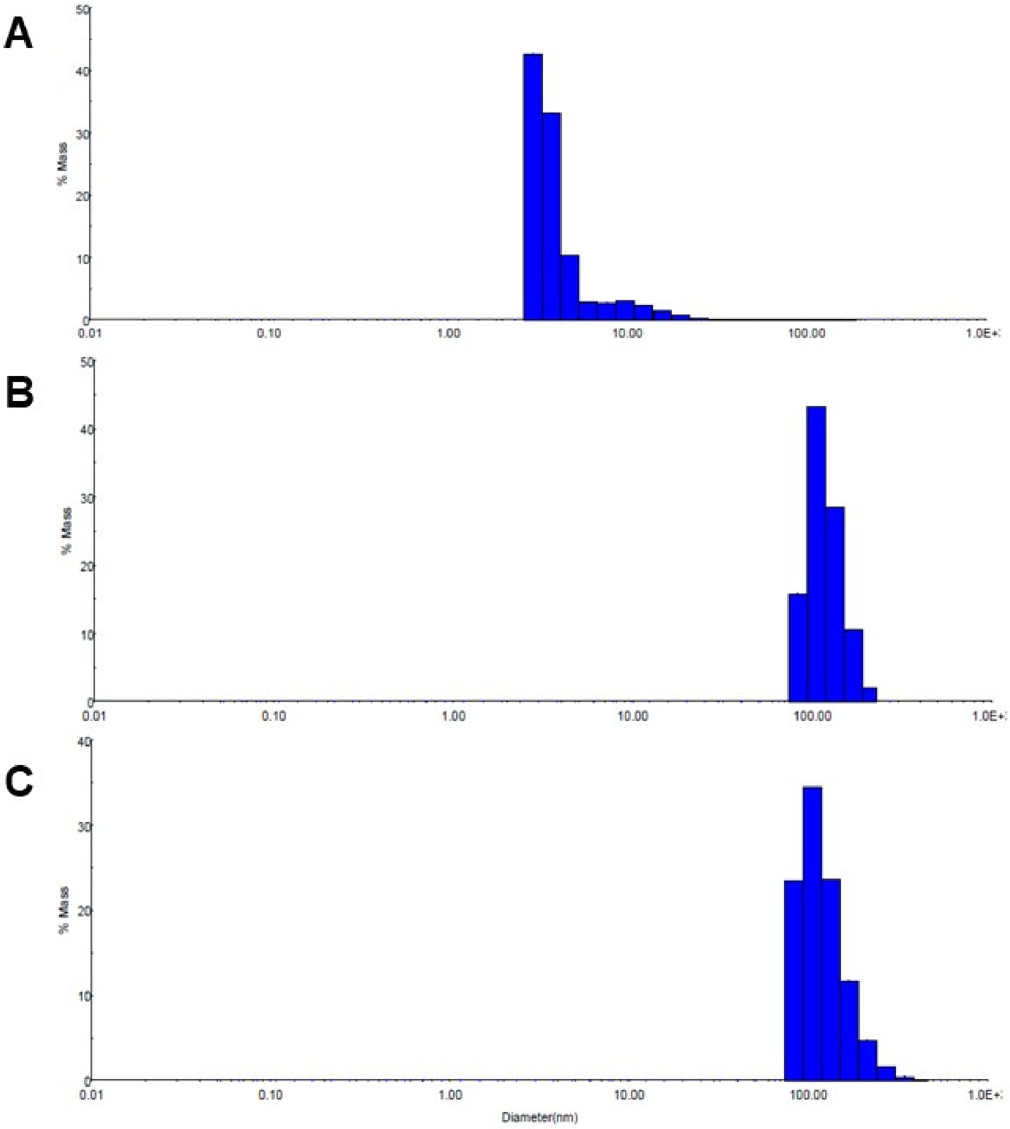

Figure Supplementary 1. Representative DLS size distribution histograms corresponding to Figure 3. A, CS. **B,** PCMal. **C,** PCMal-CS. The size values shown in Fig. 3 are obtained from the highest peak of the histogram for each sample.

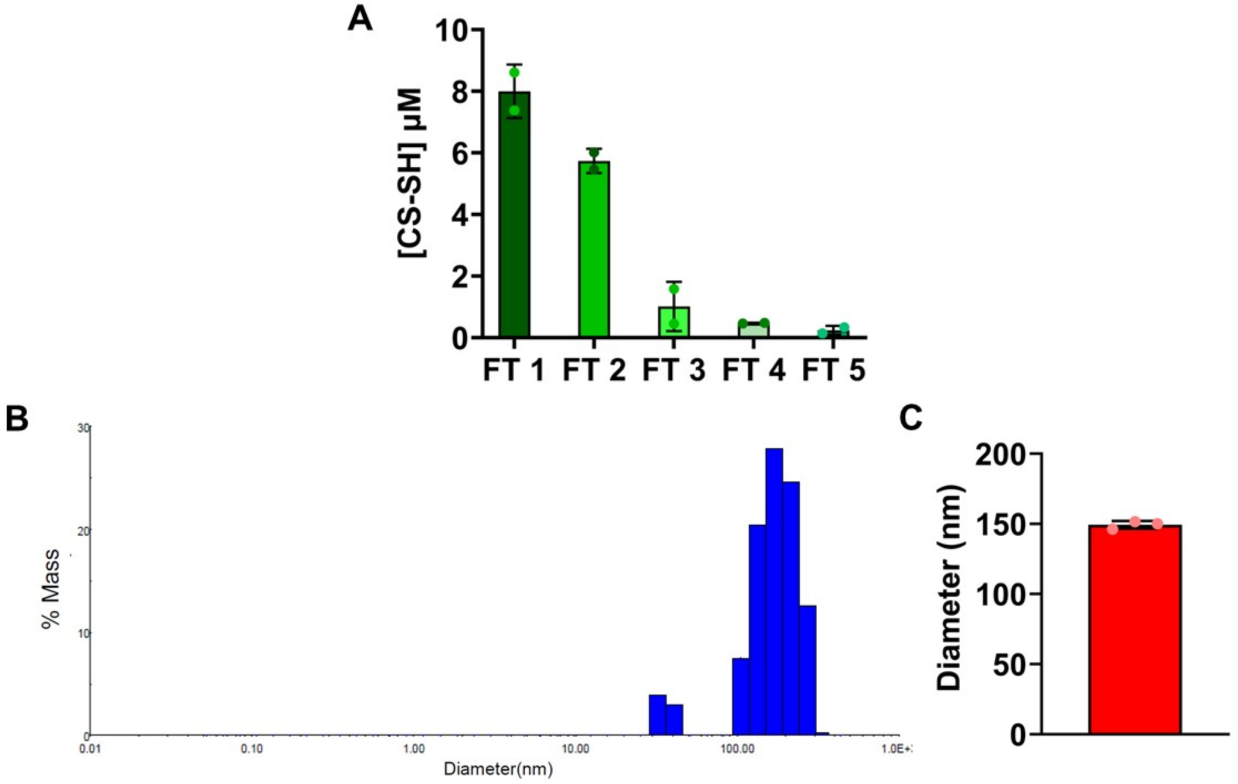

Figure Supplementary 2. Unconjugated CS removed via centricon washings. A, Five centricon washing steps were performed after the conjugation with CS. The CS concentration was measured for each flow through (FT) after fresh buffer addition. Mean values are shown, and the error bars are S.D. for n=2. **B,** DLS was used to measure vesicle after the five centricon steps. Representative DLS histogram for PCMal-CS is

shown. **C,** Average vesicle diameter of the respective histogram peak. Mean values are shown, and the error bars are S.D. for n=3.

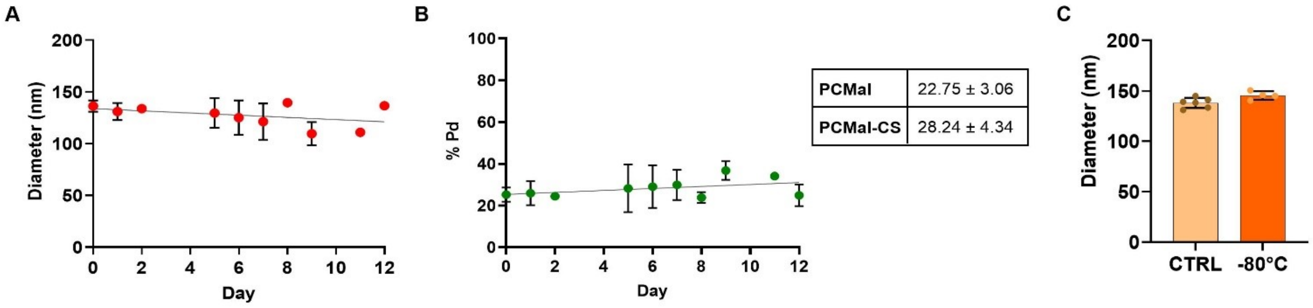

Figure Supplementary 3. DLS shows that PCMal-CS LUVs are stable. **A,** Size measurement over 12 days at 4 ºC. **B,** % of polydispersity (Pd) over the same long-term storage conditions. The table shows % Pd for vesicles before and after CS functionalization. **C,** Comparison of LUV diameter for PCMal-CS at room temperature and after a freezing step at -80 ºC. The error bars are S.D., and the fitting is a simple linear regression curve.

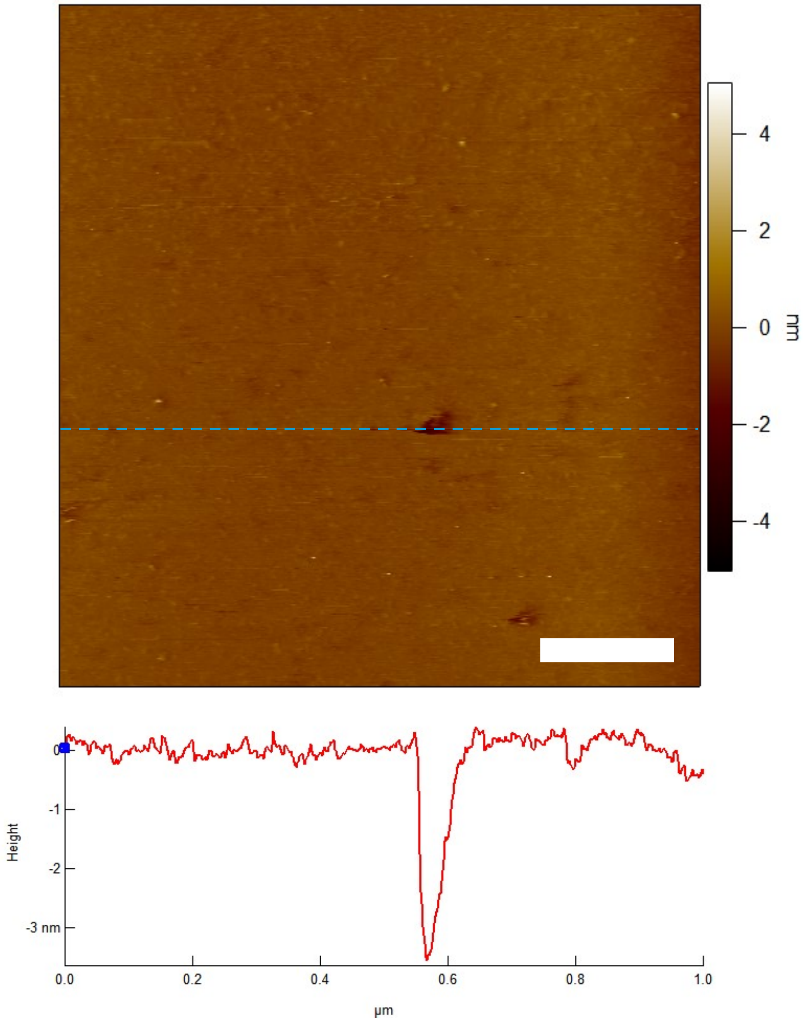

Figure Supplementary 4. Representative large-scale AFM image of supported bilayer conjugated to CS. The scale bar is 200 nm. Note that the Z-axis scale range (10 nm) is larger than in Figure 4 (3 nm). A line scan (horizontal dashed line) is used to measure height, as shown in the lower graph. The observed ~3.5 nm defect provides evidence of the presence of the membrane bilayer.

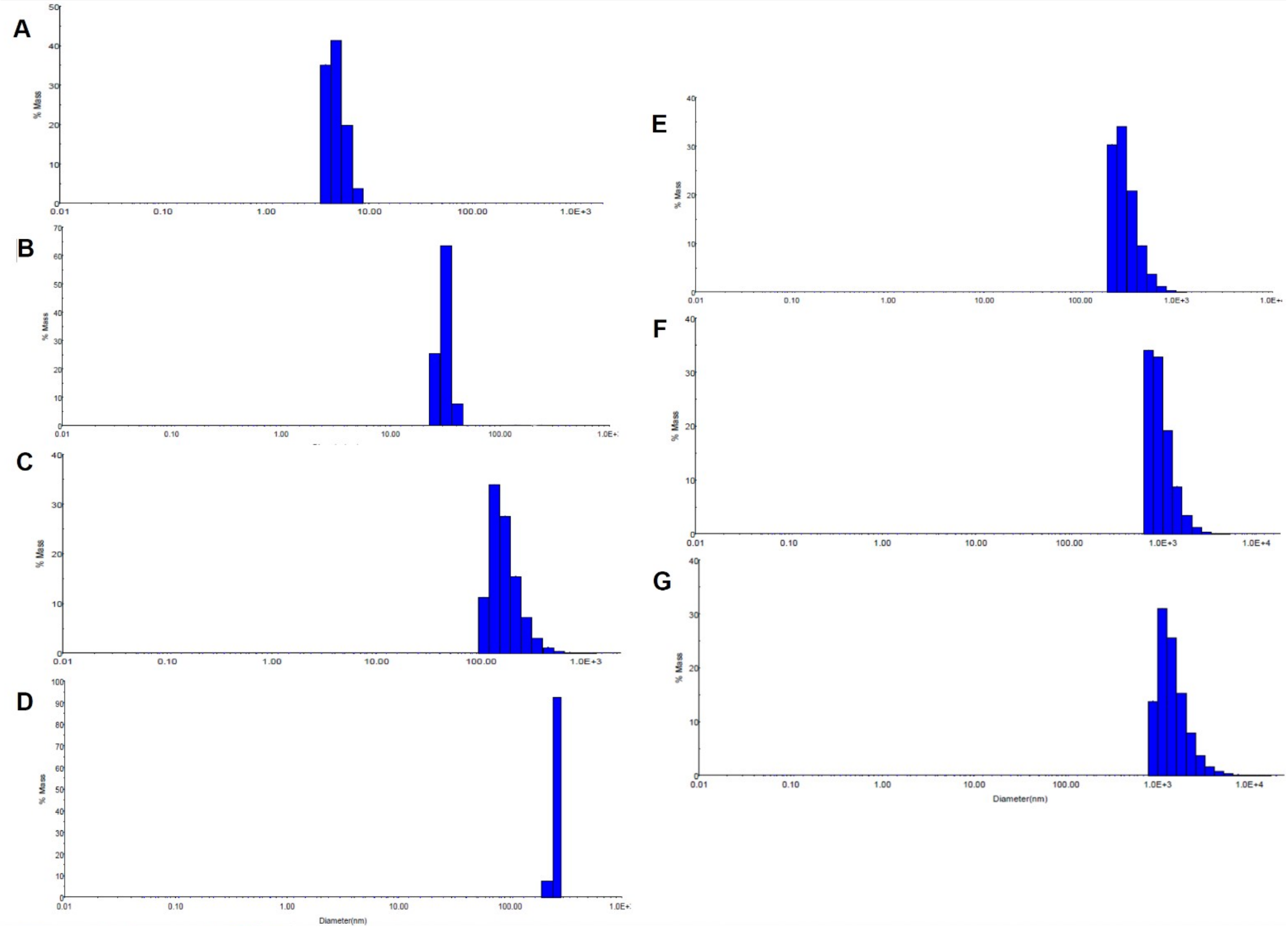

Figure Supplementary 5. Representative DLS size distribution histograms corresponding to data in Figure 5. A, PLL. **B,** PLL + CS. **C,** PCMal-CS + PLL 5 µM. **D,** PCMal-CS + PLL 10 µM. **E,** PCMal-CS + PLL 20 µM. **F,** PCMal-CS + PLL 70 µM. **G,** PCMal-CS + PLL 90 µM. The size values shown in Fig. 5 are obtained from the highest peak of the histogram for each sample.
